# Concept of neural genoarchitecture and its genomic fundament

**DOI:** 10.3389/fnana.2012.00047

**Published:** 2012-11-16

**Authors:** Luis Puelles, José L. Ferran

**Affiliations:** Department of Human Anatomy and Psychobiology, School of Medicine, University of MurciaMurcia, Spain

**Keywords:** genoarchitecture, neural Bauplan, neural gene-expression maps, neural regionalization, neural structure, progenitor domains, gene regulation networks, brain evolution

## Abstract

The recent concept of neural genoarchitecture (or genoarchitectonics) is examined from several angles, aiming to clarify the rationale for this new approach in causal and descriptive neuroanatomy. Gene expression patterns can be used as topographic stains revealing architectonic borders that may clarify, dispute, or complicate existing brain anatomical subdivisions based on other methods, while increasing our understanding of how they arise in ontogenesis and evolution. A section of the text deals with differential regulation of gene expression in an ontogenetic causal network, attending to the structure of the genome and the functional peculiarities of enhancer and repressor regulatory regions that modulate gene transcription. The emergence of regionally characteristic sets of active transcription factors represents a critical concept, molecular identity, which can be applied to discrete brain territories and neuronal populations. Gene regulation is tied to positional effects, that is, topologically invariant domains of gene expression and natural boundaries, which can be correlated with anatomic ones. The large-scale stability of these patterns among vertebrates underpins molecularly the structural brain Bauplan, and is the fundament of field homology. The study of genoarchitectonic boundaries is presented as a crucial objective of modern neuroanatomic research. At most brain regions, new neuronal populations are being detected thanks to their differential genoarchitectonic features.

## Introduction

The novel notion of *neural genoarchitecture* or *genoarchitectonics* refers to descriptions of neural structure in terms of discrete *gene expression patterns.* The core concept obviously applies as well to any other organ. This emergent anatomic approach is massively represented by diverse gene atlasing projects currently offered by the Allen Institute for Brain Science (http://mousespinal.brain-map.org), Eurexpress (http://www.eurexpress.org), or GENSAT (http://www.gensat.org), among other sources. The available data abundantly illustrate the increased dissection power of combinatorial genoarchitectonics relative to cytoarchitectony and chemoarchitectony, and the need for consequent adjustment of some conventional neuroanatomical concepts.

Such analysis has been made possible by our relatively recent technical capacity to map genes that are expressed by particular brain cells, visualizing them either as distinct cell groups, broad mantle layer domains, or entire sectors of the neural wall. This is done detecting the cytoplasmic presence of messenger ribonucleic acid (mRNA) for the selected gene product, using *in situ* hybridization (ISH) of specific antisense RNA probes (Acloque et al., 2008; Lauter et al., 2011). Individual mRNAs are transcripts of exonic sequences of genetic information (DNA). They reflect by their presence in the cytoplasm the existence of corresponding activated genes in the nucleoplasm. Depending of the activity status of the transcription promoter region found next to each gene in the genome, a number of copies of the corresponding mRNA are produced, which sort out of the cell nucleus, and associate to polyribosomes in the cytoplasm. As a result, their nucleotide sequence is translated into amino acid code, producing copies of the coded protein strands. These subsequently suffer posttranslational modification and finally acquire their functional tridimensional configurations. Many proteins remain in the cytoplasm, or are incorporated into organelles or the plasma membrane. Other proteins are secreted. However, if the proteins are transcription factors or cofactors, they return to the nucleus to interact there with DNA.

Insofar as mRNAs are nucleic acids, their detection within any tissue falls under the generic umbrella of *chemoarchitecture*. However, three features support distinguishing *genoarchitecture* from the generic chemoarchitectonic approach, that is, from non-discriminative histochemical detection of RNA: first, the *singularity* of the hybridization methodology, that specifically detects *a particular sequence of nucleotides*; second, the causal role of expressed genes and their coded proteins in *cellular typology* (represented by a set of stabile or slowly changing cell constituents, such as the cytoskeleton and differentiation-related markers) and *cellular functional state* (indicated by molecular constituents that register dynamic changes over time, either in quantity or in functional mode); third, the networked *causal role* of genomically regulated products in the conservation and morphogenetic reproduction of organic structural order, as reflected by morphostasis in phylogeny and ontogeny.

## Neural gene patterns and the morphogenetic network

A large proportion of the approximately 20,000 genes typically found in mammals are expressed in the central nervous system. Apart the so-called house-keeping genes, whose coded proteins are involved in maintaining the constituent cells alive, many other genes, which code either for *transcription factors* or for *differentiation trait proteins*, are activated or repressed in spatially restricted patterns regulated by genomic regulatory regions. These are the patterns that are the subject of genoarchitectonic studies.

Differentiation proteins, such as the enzymes synthetizing or transporting given neurotransmitters, or the respective receptors, are produced selectively in some neuronal types and brain regions. Adhesivity proteins and proteins involved in cell-to-cell communication are also regionally characteristic. The detection of the mRNA coding for them by ISH is of complementary interest with regard to immunocytochemical mapping of the proteins themselves. Occasionally, cases of non-translated mRNA are discovered (i.e., the cells have the cytoplasmic mRNA, but translation into protein is blocked, so that no corresponding protein is detected). Otherwise, ISH results are useful in cases in which cytoplasmic protein levels are very low, as occurs in immature cells, or when the protein is secreted into the surrounding intercellular matrix, or is quickly transported along the axon, since the cell bodies that synthetize the product of interest result highlighted.

The transcription factors are regulatory proteins that return to the cell nucleus, where they participate via attachment to specific DNA docking sites in positive or negative modulation of the transcription of the same or other genes. This action is often further modulated by interactions with various specific cofactor proteins; these do not attach themselves to the DNA, but form complexes with the transcription factors, altering positively or negatively their capacity to attach to the docking sites.

At variable distances from each gene promoter (upstream or downstream of the gene, or even inside the gene, for instance, inside the non-transcribed intron sequences), there appear diverse non-transcribed enhancer and repressor regulatory DNA sequences. The function of the transcription factors relates to the conditions needed for functional activation of these regulatory sequences. Efficient initiation of transcription of a gene at its promoter generally depends on the active state of an enhancer DNA sequence, whereas, alternatively, the active state of a repressor DNA sequence blocks transcription altogether. The enhancer and repressor sequences are particular stretches of DNA which contain series of assorted docking sites for several transcription factors (8 or more of them; Davidson, 2006). The characteristic tridimensional configuration of each transcription factor protein allows specific recognition and occupation of its docking site, which is the only place in the DNA where it can attach and have a regulatory effect, jointly with its specified docking companions. Note that different enhancer or repressor sequences of various genes may share a particular sort of docking site, though in each case it will be combined with a different set of other docking sites into different serial arrangements. This means that each transcription factor potentially has as many different potential modulatory functions as the number of different regulatory regions that contain at least one of its sort of docking site. A given type of docking site may be repeated side by side within the same enhancer/repressor sequence, thus implicitly requiring the attachment of two or more copies of the relevant transcription factor for the activation of the regulatory region. A crucial point is that the enhancer or repressor sequences do not become active unless their complete set of docking sites is simultaneously occupied by the relevant set of transcription factors. Partial occupancy is not functional. This condition demands the coexistence of the different sorts and quantities of needed transcription factors in the cellular nucleoplasm, by coincident production in the cytoplasm. Such coincidence only occurs in some regions of the brain, which means that the activation or repression effects are regionally restricted.

Thus, apart from a potential basal level of transcription that can be governed by the promoter by itself, both enhancement and blocking of gene transcription depends on multiple transcription factors (plus associated cofactor proteins) that happen to be present *simultaneously* in the cells of a given brain region. The variety of transcription factors present in the cellular cytoplasm, which can be modulated by epistatic intercellular signaling beyond the cell-autonomous genomic readout, results accordingly reflected after an interval in the set of gene loci that become or remain active in the cell. Adult cells tend to show only subtle changes in their gene expression patterns, in association to their dynamic functional states (plasticity). In contrast, immature developing cells will show important step-like changes in genomic readout as they differentiate, gradually developing their characteristic mature properties.

This important property of modulated genomic readout by the cells is sculpted by evolution, variously adding, deleting, or changing docking sites to individual enhancer or repressor sequences, creating new enhancers/repressors with particular sets of docking sites, or repositioning existing regulatory sequences to alternative genomic *loci* by rare recombination events. This generates diverse possibilities to influence the affected gene promoter under new conditions. It follows that the regulatory genomic regions of more evolved species typically are longer and more complex than those of less evolved animals. It is interpreted that the former have more enhancers and repressors, and can accordingly activate (or block) the same gene in more alternative circumstances and/or places of the brain primordium, thus diversifying the resulting morphogenetic outcomes (Davidson, 2006; Davidson and Erwin, 2006, 2009; Carroll, 2008).

All these phylogenetic and ontogenetic genomic aspects have to be understood under the concept of a *genetic network*. The thousands of genes present in the genome are expressed neither singly nor simultaneously, but in a specific position-dependent temporal sequence. Gene expression obviously diversifies depending on the tissue, organ, or particular cell population. The partly recursive joint functioning of such sets of genes thanks to transcription factors and cofactors, and the coexistence of epistatic effects due to concurrent intercellular signaling (via release of diffusible morphogens into the intercellular space or direct cell-to-cell contact interactions), leads to interactive effects among different parts of the genome. These represent important links in the causal network guiding morphogenesis, which includes the regulatory properties known as channeling and attractor mechanisms, and allow the manifestation of a sequential diversity of momentary equilibrium states of the whole dynamic system of cells (homeorhesis).

At each developmental stage, for any particular cell or cell population, given genes are active, whereas the rest are inactive. The active transcription factors at each embryonic locus cause the position-dependent up-regulation (activation) of some previously silent genes and the down-regulation of some previously active genes, or, in some cases, simply maintain earlier equilibrium states of gene expression. One practical difficulty is that we usually do not know precisely how many genes are involved in such effects, though analyses performed so far suggest that their number generally lies minimally in the hundreds and may go up into thousands (typical results of microchip readings with thousands of probes). The new, subtly modified active gene set performs again the same sort of interactive operations, and so on, pushing one infinitesimal step further the developing primordium toward its maturity.

Experimental embryologic data suggest that a minimum of 24 h elapse before embryonic nervous tissue changes from an earlier equilibrated state of gene expression—involving many genes—into another. However, individual changes in gene readout certainly occur in less time (even within 1 h for rapidly reacting genes), whereas others may take a longer time to occur (it depends on how we define what represents the “next” state). These mutual effects between different genomic components do not occur only in downstream direction within the conceptual genomic network chart, but generate also what amounts to recurrent upstream effects, and parallel side effects, since all regulatory regions remain open for interaction. Therefore, any causal diagram that represents how earlier states of the genetic apparatus lead to subsequent states necessarily adopts the form of a complex multidimensional network (Davidson, 2006; Davidson and Erwin, 2006, 2009). Differentiation steps imply branching of the network of all possible equilibrated states into alternative, normally spatially restricted network subregions.

There is indeed a spatial range for these phenomena, which is regulated positionally via epistatic conditions, established either by morphogens that are released from specific organizer regions and diffuse gradientally, or by mutual signaling effects between cells in contact. If epistatic (epigenetic) effects did not exist, all embryonic cells would follow the same differentiation course. The spatial range of such phenomena depends on physicochemical tissue properties (e.g., diffusion and eventual inactivation of morphogens), and is often modulated by effects of contrary sign, leading to the appearance of various patterns. Theoretical analysis suggests that short-range activation phenomena are moulded by simultaneous long-range inhibition effects (Meinhardt, 2008). There is also a time dimension to the unfolding of the morphogenetic molecular network, since the complexity of the system leads at different places and time points to the emergence of dynamic local phenomena that drive parts of the system out of partial equilibrium into a different transient equilibrium state. Such dynamics are particularly rapid at early developmental stages. The overall status of the embryonic neural system accordingly progresses along an increasingly regionalized (spatially diversified) sequence of molecularly and morphologically differentiated stages, until the relatively stable and hypercomplex adult structural state is reached.

## Relevance of relative position for genomic readout

Whereas the regulatory DNA regions of each gene implicitly contain the list of all conditions under which it can be activated or silenced, the situation in an individual cell, with a given position within the neural primordium at a specific stage of morphogenesis, will be such that probably only one enhancer or repressor sequence of all those available for any gene is activated, if at all, meaning that the cell will transcribe the gene in question or not. Extrapolation of this situation to all genes will define the genomic readout state or genetic expression profile of this cell. This does not impede any gene to be activated elsewhere (also in other organs) under the control of the same or a different enhancer. Multiple domains of gene expression (compound gene expression patterns) are observed often, indeed more frequently than unique domains. It can be determined experimentally whether a given enhancer promotes gene expression at a particular position or spatial domain in the brain primordium, by associating a transgenic reporter marker to its activation. Unfortunately, only a small proportion of the genes have been studied in this way so far.

This positional property of the regulation of gene expression is what connects the genome with developing structured morphology, and viceversa. In a curious circular logic, the sets of active transcription factors shared within characteristic spatial domains in the brain are used by the genome to activate further selective gene expression patterns at these particular positions, eventually leading to the emergence of a recognizable anatomic unit or subunit, the local *fate* detected in fate mapping studies. Evolutionarily selected unique sets of transcription factors thus in a way symbolize genomically the operationally distinct places available in the developing organ, that is, those distinguishable by the genome for proceeding with morphogenesis via specific changes in the corresponding developmental gene expression pattern. These genomically coded positions commonly represent given *neuroepithelial areas* in the early neural tube, rather than individual neuroepithelial cells or derived neurons. This explains that particular neuronal cell types are characteristically produced within a given expanse of the neural wall that has reproducible boundaries related to a characteristic genetic expression profile. Ideally, the latter involves the full set of transcription factors and cofactors that jointly define uniquely the locus. At the present state of knowledge we rarely know this full set, and thus momentarily have to define tentatively these areas with a handful of known markers (remarkably, we already can distinguish that a variety of gene markers respect the same morphological boundaries; there is no chaos in the observed patterns). Such a neuroepithelial area constitutes the relatively homogeneous *progenitor domain* for characteristic neuronal populations. Note that a progenitor domain may produce different cell types over time, due to progressive changes in its genomic readout state. Sometimes, particular bi-stable activation-inhibition dynamics of the genomic network cause two alternative specification states to emerge in a salt and pepper pattern within a progenitor domain, causing the simultaneous mixed production of two kinds of neurons (e.g., the variety of photoreceptors and other neurons that intercalate side-by-side in the neural retina).

Though a complete map of all the domains within the neural primordium that correspond to specific genomic docking site codes is not yet available, it is assumed that the mapping must be complete, that is, that all parts of the neural tube are selectively recognized and, consequently, targeted by one or several genomically regulated mechanisms, probably with substantial overlap on the whole. The existence of enhancer and repressor regulating DNA sequences suggests that the final map of identifiable progenitor domains results from the filtering overlap between activating and repressing effects. This situation would be expected, considering the aleatory and opportunistic way by which evolution has incorporated these regulatory mechanisms into the genomes.

The molecularly defined progenitor domains thus seem to represent the natural units of neural position, that is, the natural units of subsequent mature anatomy. The term *natural* applies, because they result from genomic DNA sequence, the intrinsic source of structure, in contrast with man-made, that is, *artificial*, anatomic entities. However, we still do not know how many of such natural units there are in any brain. Nevertheless, large-scale homology across vertebrates seems to apply, suggesting that the genomic network controlling neural primordia must be very conservative. We modernly understand homology (*same organ* in different species, irrespective of similarity and function) as topologic invariance both within the causal genomic network and the developing Bauplan; the invariance owes to significant conservativeness of DNA sequence copies produced across the intervening generations of descent from the common ancestors. Another pending issue is whether there are contingent rules conditioning the position, average size, and boundary properties of neural progenitor domains.

The individual progenitor domains are not necessarily specified separately one by one, since often differential genomic readout depends on relative cellular position within epistatic field effects (influenced by neighboring cells), with simultaneous reaction of many cells within an ample field to varying concentrations of diffusing morphogens (the spatially restricted sources of these signals are known as *secondary organizers*). The relative amount of morphogen signal detected by any cell within the limits of its sensitivity is variously translated according to its competence (given by its previous genomic equilibrium state) into different transcription factor codes; we observe normally a step-like readout function of the efficient signal gradient within several neuroepithelial areas increasingly distant from the signal source. This leads to the side-by-side generation of a set of distinct progenitor domains with differential molecular profiles within a larger initially multipotent territory. The concept of *morphogenetic field* has been thought to apply in such self-regulated complex domains of secondary patterning within a developing primordium (Puelles and Medina, 2002; Medina, 2007; Bardet et al., 2010).

For instance, it has been shown that the whole hindbrain alar plate is competent to react to Fgf8 signals in order to produce characteristic cerebellar structures (Martínez et al., 1995). Normally, only rostral hindbrain areas receive significant levels of such signals, which are released from the isthmic secondary organizer at the rostral end of the hindbrain. Therefore, only a restricted alar subregion fulfills its cerebellar potency, causing the cerebellum to form behind the isthmus, and no cerebellum appears more caudally. However, similarly potent hindbrain areas lying even closer to the isthmic organizer do not form a cerebellum, and produce isthmic nuclei instead. A higher level of the efficient signal found at this locus thus results in an alternative fate, that is, in an alternative progenitor domain with a different structural fate of its derivatives.

Analysis of such epistatic position-dependent secondary patterning effects suggests that there are at least three distinct levels of genomic regulation of neural anatomic complexity, in which cell-autonomous and epistatic position-detecting mechanisms are mixed. The first level establishes *neural identity* generally, as well as a primary variety of regional flavors, in the earliest primordium, the neural plate (Puelles et al., 2005). Spatial molecular heterogeneity at this level is affected by the *primary organizer* signal sources (e.g., the node and the rostral visceral endoderm, as well as the prospective roof plate and floor plate domains and related vertical inducing tissues, such as the notochord and non-neural ectoderm). Planar and vertical signaling causes superposed epistatic effects upon ongoing cell-autonomous transcription factor regulation, leading to differential emergence of transient (dynamic) regional genoarchitectonic patterns, whose boundaries depend on their relative initial positions. Note that the early neural primordium registers substantial surface growth (Sanchez-Arrones et al., 2009, 2012), as well as elongation and neurulation shape changes, which continuously modify the positional readout of diffusing or cell-cell contact-mediated molecular signals. As a result, the second regulatory level is represented in the early neural tube by a spatially distributed semi-stable set of primary molecular identities within the early neuroepithelium (initial or *primary AP and DV regionalization*). This confers a range of cell-autonomous broad differential potencies, and causes the secondary organizers to emerge via interactive cell-cell communication at relevant borders. Signaling from these new organizers drives the appearance of the subsequent third regulatory level, implying *field patterning* effects, by the effects of novel morphogens that spread gradientally and allow individual smaller neuroepithelial areas receiving these signals to choose differentially among their initial potencies according to relative local signal strengths, thus giving rise to the definitive or *secondary molecular identities* of the progenitor domains. Such three-fold specification of the progenitor domains is followed by triggering of selective areal histogenetic patterns (e.g., a differential program for proliferation, neurogenesis, gliogenesis, neuronal migration, and stratification at each progenitor domain).

The fact that regionalization of the neural primordium progresses from few relatively large initial territories (distinguishable both molecularly and via fate mapping in the neural plate; Puelles et al., 2005; Sanchez-Arrones et al., 2009, 2012) to smaller subsequent subdivisions (brain vesicles, neuromeres, segmental DV regions, microzones, individual progenitor domains) reveals that aspects of relative timing are also relevant in this ontogenetic mechanism. As expected, some genes begin or cease to be expressed once the developmental process has reached given stages.

## Areal molecular identity and its developmental consequences

The combined set of transcriptor factors activated at any particular neuroepithelial areal domain progressively drives via its interaction with genomic regulatory regions the local histogenesis and morphogenesis. Note that *histogenesis* refers to cellular differentiation and migration phenomena, including axonal navigation and synaptogenesis, whereas *morphogenesis* alludes to macroscopic supracellular phenomena, such as thickening or bending of the neural wall. Such downstream developmental consequences of a specific molecular identity occur separately within each natural domain of the neuroepithelium that has been differentially specified molecularly, independently from what happens at neighboring domains, unless epistatic morphogenetic field effects that mutually correlate neighboring behaviors are involved (Guillemot, 2007). Areal anatomic delimitation probably cannot be expected before definitive molecular identities exist in a given neural territory.

**Figure 1:**
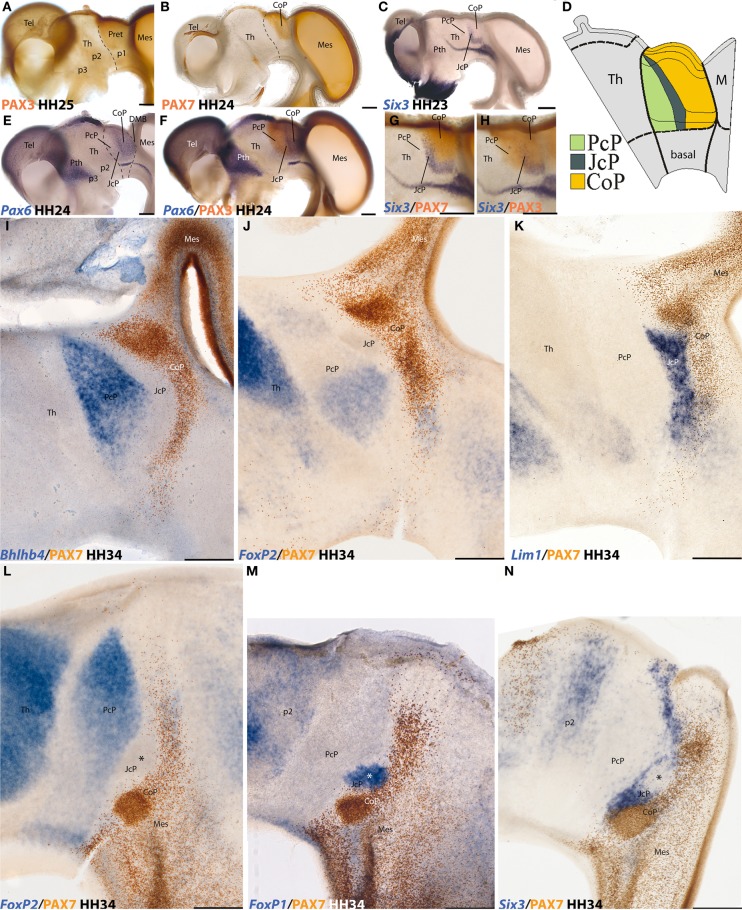
**Examples of neural genoarchitectonic analysis of the pretectal region in the chick brain, based on data reported by Ferran et al., 2007, 2009.** Panels **(A–H)** show half-brain whole-mounts of embryonic chick brains at 4–5 days of incubation (HH stages are indicated), immuno-reacted or *in situ* reacted for various transcription factor genes that establish boundaries defining the pretectum and its main inner subdivisions. *Pax3* marks the pretecto-thalamic border **(A)**, also drawn in **(B)**. *Pax7* is restricted to a caudal pretectal domain, identified as “commissural pretectum” (CoP in **B**). *Six3* labels a thin intermediate pretectal sector, named “juxtacommissural pretectum” (JcP in **C**). Rostral to JcP there remains a third sector adjacent to the thalamus, known as “precommissural pretectum” (PcP in **C**). A schema of PcP, JcP, and CoP appears in **(D)**, with a color-code. Note the pretectum is a well-defined alar domain limited rostrally by the thalamus (at left in **D**) and caudally by the midbrain (at the right in **D**). Thinner secondary lines define dorsoventral subdomains delineated in the pretectum by these or other markers **(D)**. The *Pax6* pattern in **(E)** is also restricted to CoP (as *Pax7* in **B**), but extends ventrally all the way to the alar-basal boundary. Note JcP and PcP are unlabeled in **(E)**, but double labeling with *Pax6* and *Pax3* in **(F)** reveals their position; note *Pax3* does not extend as much ventrally as *Pax6* (compare DV limits in **D**). **(G,H)** Comparison of the intermediate JcP pattern of *Six3* with the CoP domain defined by *Pax7* **(G)** and with the whole pretectum marked by *Pax3* **(H)**. All these distinct areas are interpreted as differentially coded progenitor areas likely to produce different neuronal populations. At the stages shown neurogenesis is already in progress. The panels **(I–N)** show mapped thick sections through the avian pretectum at 8 days of incubation (HH34), divided into 3 sagittal sections **(I–K)** and 3 horizontal sections **(L–N)**. In all cases two markers are labeled either in blue (*in situ*) or in brown (immunoreaction) (see lower left corner for marker identification). The observed expressions largely pertain to postmitotic neurons born at the previously demonstrated PcP, JcP, and CoP pretectal domains, which are now differentiating in the mantle layer, superficial to their original progenitors. Note that the respective boundaries between the three domains are well conserved (PcP, blue, JcP, unlabeled, CoP, brown in **I, J,** and **L**), and individual nuclei start to be recognizable (not identified in these images). The molecular properties of the derivatives of each domain extend from periventricular to subpial levels of the mantle. The JcP domain is labeled differentially from the CoP (**K** and **N**). The asterisk within the JcP domain in N marks a restricted cell group that secondarily loses the *Six3* marker, typical of JcP, and up-regulates instead the *FoxP1* marker **(M)**.

The component cells of these differentiating spatial domains (both neuroepithelial matrix cells—the progenitors—and derived postmitotic neurons and glia cells) jointly keep activated the enhancers of specific genes, thus producing a shared gene expression profile (e.g., see Figure 1). Such a state can be transient or permanent, and is currently understood as the domain's *molecular identity*. As development proceeds, domain subdivisions may appear, distinguished by alternative network equilibrium states of the respective molecular codes. These ulterior changes in genomic state may result cell-autonomously from subtle readout in terms of nascent pattern of earlier gradients of endogenous signals, or from further epistatic cell-cell signaling (new emergent organizers), leading eventually to further steps in regionalization.

The *natural boundaries* of these genomically coded domains accordingly are the real causal boundaries underlying morphogenesis and anatomy, since *all morphogenesis is an epiphenomenon of underlying and contextual histogenesis*. Sometimes neuroanatomical tradition has already identified with more or less precision some of these natural brain boundaries detected genoarchitectonically. Classic success generally occurred at sites where it was obvious that the cell fates at both sides of the boundary are quite different. Common sense already tells us that whenever things are systematically different across a line, it occurs because the line is a boundary between domains with differential causal histories. Sometimes embryologists, professionally closer to the causal mechanisms, noted the natural boundary, but other colleagues preferred to follow contrary anatomic traditions derived from adult description. A good example of such discrepancy is provided by the classic plane delimiting midbrain from diencephalon across the posterior commissure, supposedly ending at the mamillary body, which is copied in many textbooks. This boundary was tentatively suggested by His (1893), acknowledging lack of any anatomical basis for it, and justifying it merely as an arbitrary provisional reference until an appropriate boundary could be defined. Although the natural developmental boundary consistent with current genoarchitectonic data (Ferran et al., 2007, 2008, 2009; i.e., a plane passing transversally *behind* the posterior commissure and just in front of the oculomotor nerve root, far from the mamillary pouch; Figure 1) was already postulated by Rendahl (1924) using mere hematoxylin cytoarchitectony, very few anatomists followed his embryologic cytoarchitectonic rationale, and most retained as a dogma the unsupported proposal of His (1893). In some other cases, the study of genoarchitectural patterns surprises us with unexpected limits, hitherto wholly unrecognized, which illuminate the postulated traditional boundaries as not being consistent with the natural ones (e.g., Shimamura et al., 1995; Ferran et al., 2007, 2008, 2009; Marín et al., 2008; Thompson et al., 2008; Shimogori et al., 2010; Medina and Abellan, 2011; Merchan et al., 2011; Morales-Delgado et al., 2011; Alonso et al., 2012; Lorente-Cánovas et al., 2012; Puelles et al., 2012a,b).

We defend that advanced understanding of brain morphogenesis and adult structure lies, far from considering neuroanatomy finished with the foregoing millenium, in continuing to develop conceptual anatomic *models* whose postulated boundaries are increasingly consistent with the natural genomically recognized genoarchitectonic boundaries. The rationale is simple: these relate directly to the causal mechanisms that create the relevant morphologic subdivisions, whereas other boundaries are arbitrary.

For instance, there is no point now in following the anatomical tradition that includes the pretectum in the midbrain, since the differential genomic regulation of the pretectum and midbrain identities distinguishes these territories as having causally independent and incompatible fates [there are mutual repressive effects between their respective molecular backgrounds; see discussion in Ferran et al. (2007)]. As a conclusion, we regard now the pretectum as an independent spatial region of the diencephalon—it represents a caudal part of the diencephalic field—having a distinct genoarchitectonic identity code. This does not impede that this region shows a distinct checker-board pattern of component anteroposterior and dorsoventral progenitor domains and subdomains, leading to the spatially stereotyped production of neurons for about 30 different pretectal populations (Figure 1; Ferran et al., 2007, 2008, 2009; Merchan et al., 2011). Genoarchitecture and related developmental patterning accordingly represent crucial guides in our selection of significant anatomic boundaries and structural understanding.

One important function of the neural genoarchitectonic approach therefore is to illuminate novel details in this general histogenetic scenario and provide evidence supporting the definition of natural (i.e., genomic-underpinned) boundaries. These newly emerging complexities of neural anatomy will substitute profitably the rough boundaries (when not fully erroneous boundaries) produced by earlier cruder approaches (e.g., using gross morphology, adult dissection, ventricular sulci, or cytoarchitecture).

## Evolutionary aspects of the genoarchitectonic neural bauplan

If we ask about what lies behind the apparently haphazard expression of thousands of genes here or there in the brain, the answer is that certainly it was at bottom blind variation and natural selection what eventually constructed the extant genomes over millions of years. These slow mechanisms lack any intentional or teleological logic, that is, any design, even though the surrounding nature may have exerted contextual constraints that contributed historically to its precise shaping. The genes and their regulatory sequences evolved, and the expression patterns we observe emerged, as animals with brains appeared, leading to a variety of functional brains that, integrated in the similarly evolving bodies, were all compatible at least with transient survival in the correspondingly changing environmental conditions. Selected genomic configurations promoting population-wise biological fitness were perpetuated conservatively by the precise DNA duplicating machinery.

Nevertheless, the absence of a preconceived design does not mean there is absolute chaos in the genome, or in our brains. A measure of order is provided by the widespread conservation of the expression pattern topology of neural genes across all vertebrates, as well as by more or less evident links with some invertebrate gene patterns (e.g., rostrocaudal colinearity of *Hox* genes). This bespeaks of comparable, even if not identical, attractor mechanisms in the relevant developmental processes and resulting morphogenesis. The causal gene network has a cybernetic structure, whose deviations seem to become efficiently buffered via numerous corrective mechanisms that act against accidental change.

The evolution of life forms necessarily had to proceed stepwise via variation and selection from more or less fit antecedent states to “more fit” consequent ones. Each generation must have been at least minimally fit under local conditions, since the ancestors did have descendants. The descendants slowly evolved along the process into divergent forms, each adapted for survival within the range of available environmental niches. Among a diversity of invertebrate animal forms, a particular lineage evolved vertebrate features, a major evolutionary step possibly helped by an antecedent genomic duplication. Genomic duplications are advantageous for evolution, since variations are allowed in the newly available gene copies, whereas survival remains warranted by unchanged ones. This led to the evolutionary radiation of vertebrate animal forms, in which some additional whole genomic duplications occurred, as well as isolated duplications or recombinations of given genes or gene groups (Ohno, 1970; Blomme et al., 2006; Carroll, 2008). The respective genomes of the extant vertebrate forms reflect in their fundamental structure the shared ancestral antecedents, jointly with evidence of the rare genomic duplication events, as well as of other non-shared evolutionary modifications accrued in specific lineages. The changes affect mostly the DNA regulatory regions, rather than protein-coding sequences.

The exons of genes (the portions that get transcribed and eventually are translated into protein sequence) are highly conserved, even between invertebrates and vertebrates, particularly at the places where *functional protein motifs* are coded, that is, the aminoacid sequence subregions essential for protein interactions in characteristic functional contexts. In contrast, the introns and the equally non-translated regulatory DNA sequences flanking the genes tend to increase in number and variety along the course of evolution. Interestingly, some of these regulatory sequences are also characteristically conserved along diversified evolutionary radiations. Some non-translated regulatory sequences are found at long genomic distances from the promoters they interact with. Spatial buckling of the DNA strands, controlled by specific proteins, is held to underlie such distant regulatory effects (modification of DNA buckling is another way by which genomic regulation has evolved over time). It apparently is easier, or statistically more efficient, and less dangerous for fitness, to add optional regulators, than to modify or translocate existing ones.

Any genomic variation in the genes themselves or in their regulatory regions that statistically improved fitness of the progeny was opportunistically retained by recruitment into existing regulatory networks, and eventually probably contributed to consequent further evolution (diversified growth of the network). Therefore, in essence we have the present structure of brain parts (over 2500 anatomic brain entities in contemporary ontologies), and the corresponding complex gene patterns, because both evolved from earlier simpler versions, always presumably as a consequence of contingent empiric improvements in adaptive fitness under particular environmental conditions (we refer here to timing in the range of millions of years). The evolutionary logic is therefore that of *survival* and *accumulation*: anything goes, in principle, but once a satisfactory solution for survival is found, it is kept and can be modified and eventually perfected ulteriorly. Widely shared aspects of animal genomes are likely to relate indirectly to unchanging properties of the world in which they have evolved. For instance, the existence of water, light, an atmosphere, and a particular range of temperatures on Earth are surely assumed by all genomes.

Evolution is therefore opportunistic in adding genomic details, but the triumph of genomes in the unlikely adventure of creating ever more complex living forms lies in the combination of their strong intrinsic tendency to structural conservativeness with a modular structure of operons that allows alternative regulatory options to be incorporated and tested. This accounts for persistent reflection of earlier efficient stages of genomic and morphogenetic structural complexity, modulated by the slowly changing epigenetic biosphere, specific adaptations, and sex-selection events developed within the animal group and, not least importantly, the practical consequences of wholly unpredictable emergent epistatic phenomena (e.g., the emergence of flight in insects, allowing a novel way of interacting with the world around, or the development of unique social capabilities in hominids; Amis (2008) notably included among the latter: conversation, hilarity, and drinking).

The statistically persistent regularities of our world over evolutionary time have conditioned the genomes to stabilize (or reproduce repeatedly via parallel evolution) the developmental production of characteristic gene activity patterns in the brain primordium. Whatever simple brain structure first evolved in the earliest vertebrates has conditioned subsequent brain structure heavily. A major change with respect to invertebrates was the emergence of the neural tube as a non-ganglionic central nervous system—we are not only vertebrates but also cerebrates. Conservative evolution is possibly stronger in the brain than in other body organs, due to the constrained need of complex functional interaction among its multiple parts for efficient signal processing and internal map production, and consequent systemic homeostasis and survival in the world. Whereas entire limbs may come and go (snakes, whales), and hearts can operate similarly being bi-, tri-, or tetra-chambered, tested brain modules probably are scarcely modifiable, and may not be easily replaceable in practice by completely different designs. Against conceptions of brain evolution postulating successive addition of brain parts (e.g., the triune brain theory of MacLean, 1990), there is now a wide consensus on the idea that most brain parts of gnathostomes are already present in the simplest extant forms, and many of them also appear in agnathans (review in Puelles, 2001).

Instead, the evolving genomes have had opportunity to incorporate multiple change-buffering and redundant mechanisms which insure that, independently of changing circumstances, particular neuronal populations, neural pathways, and tested functional synaptic connections are correctly produced. This means securing the existence of appropriately positioned and molecularly specified neural progenitor areas (and the associated morphogenetic fields). This change-buffering tendency of evolving genomes has been identified as evolutionary genomic *channeling* (Waddington, 1975), or as sculpturing of the morphogenetic landscape into *attractor fields* (Striedter, 1998).

Such genomic channeling amounts collectively to constrained development and leads to the emergence of a Bauplan, that is, of an organized and characteristic *invariant set of morphogenetic and structural features* which are shared among evolutionarily related animal forms (simplesiomorphies). Such morphostatic genome-controlled aspect of brain morphogenesis is the fundament of neural *homology*, that is, of true “sameness” relationships across the brains of diverse species, which often has to be understood as field homology (Puelles and Medina, 2002; Medina, 2007).

The root of the structural Bauplan for vertebrate brains apparently lies at the transition of agnatha to gnathostomes, since the brain of the former is divergent or incomplete in some aspects (Puelles, 2001; Pombal et al., 2009). Unfortunately, there is still very little information about gene expression patterns in the brain of agnatha. In contrast, fundamental neural genoarchitecture essentially has changed minimally (hardly at all) since the appearance of cartilaginous fishes, irrespective of ulterior adaptations to various niches in the sea and to non-aquatic modes of life. The ulterior emergence in mammals of a six-layered neocortex, a more complex thalamus, a perfected hippocampal cortex, and a claustro-amygdaloid complex, as well as other specific neural adaptations in other vertebrate lineages, represented significant elaborations of the original Bauplan with changes that apparently are more quantitative than qualitative (Holmgren, 1922, 1925; Puelles, 1995, 2001; Nieuwenhuys et al., 1998; Nieuwenhuys, 2009).

### Conflict of interest statement

The authors declare that the research was conducted in the absence of any commercial or financial relationships that could be construed as a potential conflict of interest.
